# *Terminalia chebula* Extract Protects OGD-R Induced PC12 Cell Death and Inhibits LPS Induced Microglia Activation

**DOI:** 10.3390/molecules18033529

**Published:** 2013-03-19

**Authors:** Bhakta Prasad Gaire, Nirmala Jamarkattel-Pandit, Donghun Lee, Jungbin Song, Ji Young Kim, Juyeon Park, Soyoung Jung, Ho-Young Choi, Hocheol Kim

**Affiliations:** 1 Department of Herbal Pharmacology, Kyung Hee University, College of Oriental Medicine, Seoul 130-701, Korea; 2 Korea Institute of Science and Technology for Eastern Medicine (KISTEM), NeuMed Inc., Seoul 130-701, Korea

**Keywords:** *Terminalia chebula*, oxygen-glucose deprivation, PC12 cells, ischemia

## Abstract

*Terminalia chebula*, native to Southeast Asia, is a popular medicinal plant in Ayurveda. It has been previously reported to have strong antioxidant and anti-inflammatory efficacy. In this study, we aimed to investigate if fruit extract from *T. chebula* might protect neuronal cells against ischemia and related diseases by reduction of oxidative damage and inflammation in rat pheochromocytoma cells (PC12) using *in vitro* oxygen-glucose deprivation followed by reoxygenation (OGD-R) ischemia and hydrogen peroxide (H_2_O_2_) induced cell death. Cell survival was evaluated by a 2-(4,5-dimethylthiazol-2-yl)-2,5-diphenyltetrazolium bromide (MTT) assay. Free radical scavenging, lipid peroxidation and nitric oxide inhibition were measured by diphenyl-1-picrylhydrazyl (DPPH), thiobarbituric acid (TBA) and Griess reagent, respectively. We found that *T. chebula* extract: (1) increases the survival of cells subjected to OGD-R by 68%, and H_2_O_2_ by 91.4%; (2) scavenges the DPPH free radical by 96% and decreases malondialdehyde (MDA) levels from 237.0 ± 15.2% to 93.7 ± 2.2%; (3) reduces NO production and death rate of microglia cells stimulated by lipopolysaccharide (LPS). These results suggest that *T. chebula* extract has the potential as a natural herbal medicine, to protect the cells from ischemic damage and the possible mechanism might be the inhibition of oxidative and inflammatory processes.

## 1. Introduction

Natural antioxidants, from medicinal or edible plants, have recently received much attention as promising agents for reducing the risk of oxidative stress-induced neurological diseases [1,2]. Reactive oxygen species (ROS) and reactive nitrogen species (RNS) are the main source of free radicals, and they lead to serious damage causing neurodegeneration in the pathogenesis of neurological disorders such as Alzheimer’s disease, Parkinson’s disease and strokes [3,4,5]. Overproduction of ROS, during ischemia/reperfusion, causes an imbalance between oxidative and anti-oxidative processes leading to mitochondrial dysfunction, excitotoxicity, lipid peroxidation, and inflammation. Therapeutic efforts aimed to counteract ROS or inhibit their formation, have been shown to be beneficial in various neuro-degenerative diseases, including ischemia [6].

*Terminalia chebula* Retz*.* (Combretaceae), commonly known as chebulic myrobalan, is a popular medicinal plant in the Ayurvedic system of medicine. The main compounds from *T. chebula*, such as gallic acid, ellagic acid, chebulanin, chebulinic acid, rutin casuarinin, and quercetin, were reported to possess antioxidant effects [7,8]. Traditionally, *T. chebula* has been used to treat kidney and urinary disorders, nervous disorders, colic pain, chronic cough, sore throat, asthma, *etc*. It is also used as laxative, antitussive, diuretic, digestive, antidiabetic, and as a cardiotonic remedy [9,10]. *T. chebula* has been reported to exhibit anticancer [11], antidiabetic [12], antimutagenic [13], antibacterial [14] and cardio-protective activities [15]. *T. chebula* is also reported to possess strong anti-inflammatory activity [16].

Based on the information regarding antioxidant and anti-inflammatory effects, we hypothesized that fruit of *T. chebula* extract may have protective effects on neuronal cell death against ischemia and related diseases. In this study, we aimed to investigate the protective effect of *T. chebula* extract against OGD-R and hydrogen peroxide (H_2_O_2_) induced cell injury on the rat pheochromocytoma cell line (PC12 cells). The reason for choosing PC12 cells is that, they display phenotypic characteristics of adrenal chromaffin cells and sympathetic neurons and therefore, PC12 cells are a well-known model for neuronal differentiation [17,18]. OGD-R, a well-accepted *in vitro* ischemia model [19,20], was used to evaluate the protective effect of *T. chebula* extract. H_2_O_2_ induced cell death; an extensively used *in vitro* oxidative stress model [21,22], was used to evaluate the effect of *T. chebula* extract in PC12 cells. Furthermore, the activation of the primary microglia is one of the major reasons of cellular inflammation in ischemic condition and lipopolysaccharide (LPS) activated BV2 cells are reported as the suitable alternatives for primary microglia in the culture or animal experiments [23]. Therefore, we investigated the inhibitory effect of *T. chebula* extract on LPS activated microglia cells (BV2 cell). We also determined the diphenyl-1-picrylhydrazyl (DPPH) free radical scavenging, lipid peroxidation and nitric oxide (NO) inhibition activity of *T. chebula* extract.

## 2. Results

### 2.1. HPLC Analysis and Total Phenol Content of T. chebula Extract

**Figure 1:**
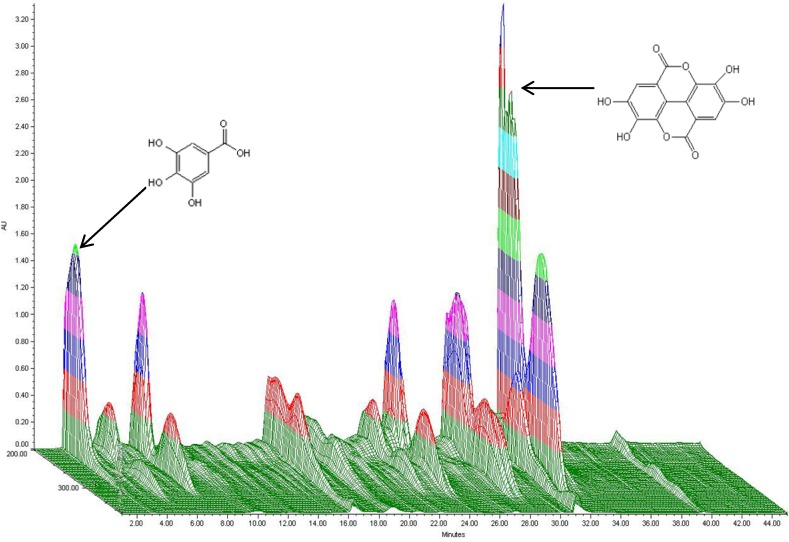
3D HPLC spectrum of *T. chebula* extract showing gallic acid and ellagic acid.

The 3D HPLC spectrum of *T. chebula* extract is shown in Figure 1. The major polyphenols isolated from the *T. chebula* extract were gallic acid and ellagic acid.

### 2.2. DPPH Radical Scavenging Activity of T. chebula Extract

*T. chebula* extract showed dose dependent radical scavenging activities with a maximum of 96% inhibition against DPPH radical at 100 μg/mL. The effective concentration of *T. chebula* extract to scavenge 50% of DPPH free radical (EC_50_) was found as 5.5 ± 1.1 μg/mL. Ascorbic acid (EC_50_ of 4.7 ± 0.01 μg/mL) was used as a positive control. Table 1 showed the content of gallic acid, ellagic acid and total phenols in *T. chebula* extract.

**Table 1:** Total phenols gallic and ellagic acid yield from *T. chebula* extract.

Total Phenol (mg GAE/g)	Gallic acid (mg/g)	Ellagic acid (mg/g)
787.1 ± 20.8	29.8 ± 2.8	25.5 ± 2.2

### 2.3. Effect of T. chebula Extract in PC12 Cells against OGD-R Induced Cell Death

**Figure 2:**
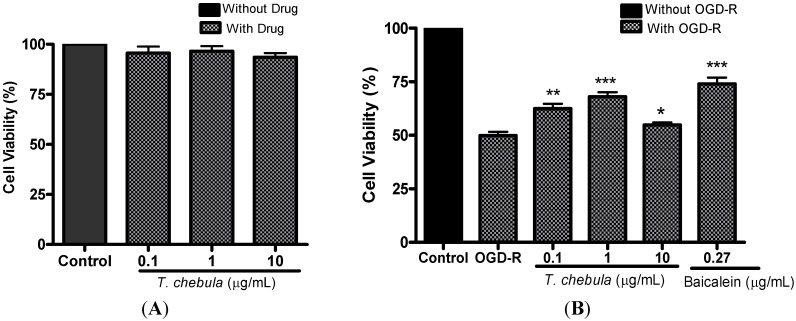
Effect of *T. chebula* extract on PC12 cells. (**A**) Cytotoxic effect of *T. chebula* extract on PC12 cells. Different concentrations of *T. chebula* extract were treated on PC12 cells for 28 h in normal condition. (**B**) Effect of *T. chebula* extract against OGD-R induced cell death on PC12 cells. PC12 cells were exposed to OGD for 4 h followed by re-oxygenation for 24 h. Different concentrations of *T. chebula* extract were treated 30 min before and during 4 h of OGD. PC12 cells viability was measured by MTT assay. Control group served as 100%, and data obtained in other groups were calculated as percentage of control accordingly. * *p <* 0.05, ** *p <* 0.01 and *** *p <* 0.001.

The survival of cells after treatment of *T. chebula* extract was measured by an MTT assay. *T. chebula* extract (at 0.1–10 μg/mL) didn’t show any cytotoxic effects on PC12 cells (Figure 2A**)**. To examine whether *T. chebula* extract protects against OGD-R induced cell death, PC12 cells were incubated without glucose solution in the hypoxia chamber for 4 h followed by 24 h of reoxygenation with glucose solution in normal incubator. Different concentrations of *T. chebula* extract were treated 30 min before and during 4 h OGD. Cell viability in OGD group was 50.4 ± 1.5% whereas cell viability in *T. chebula* extract treated cells (at 0.1, 1, 10 μg/mL) were 62.4 ± 2.2% (*p* < 0.05), 68.0 ± 2.0% (*p* < 0.01), and 54.8 ± 1.2% (*p* < 0.05) respectively, as compared to the control group (set 100%). Baicalein at 0.27 μg/mL (1 μM) treated cells showed 74.0 ± 2.8% (*p* < 0.001) cell viability against OGD-R (Figure 2B).

### 2.4. Effect of T. chebula Extract in H_2_O_2_ Treated PC12 Cells

**Figure 3:**
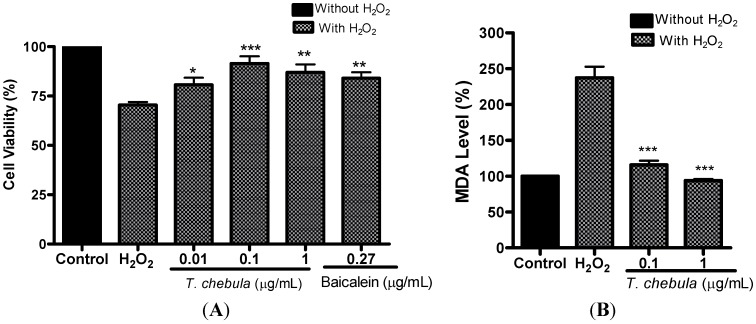
Effect of *T. chebula* extract on H_2_O_2_-induced cell death and lipid peroxidation assay. PC12 cells were exposed to 200 μM H_2_O_2_ for 24 h. Different concentrations of *T. chebula* extract were treated 2 h before and during H_2_O_2_ exposure. Control group served as 100%, and data obtained in other groups were calculated as percentage of control accordingly. (**A**) PC12 cells viability was measured by MTT assay. (**B**) Lipid peroxidation assay (MDA level) was measured by thiobarbutic assay. ** *p <* 0.01, and *** *p <* 0.001.

To determine whether *T. chebula* extract protects cells against oxidative stress induced cell death, PC12 cells were exposed to H_2_O_2_ for 24 h_._ The toxicity of H_2_O_2_ in PC12 cells was found to be concentration and time-dependent (data not shown). Cell viability in the H_2_O_2_ (200 μM) treated group was 70.4 ± 1.5% and that in *T. chebula* extract treated cells (at 0.01, 0.1, 1 μg/mL) was 80.6 ± 3.6% (*p* < 0.05), 91.4 ± 3.6% (*p* < 0.005), and 86.9 ± 4.4% (*p* < 0.01) respectively, as compared to the control group (100%) (Figure 3A). Baicalein at 0.27 μg/mL (1 μM) treated cells showed 84.0 ± 2.9% (*p* < 0.05) cell viability against H_2_O_2_. Previous study [24] was reported that methanol extract of *T. chebula* at 0.5 μM showed 100.8 ± 9.2% (*p* < 0.01) cell viability.

### 2.5. Effect of T. chebula Extract in Lipid Peroxidation

The TBA colorimetric assay was performed to determine whether *T. chebula* extract can inhibit the formation of MDA induced by H_2_O_2_. The cells treated with H_2_O_2_ significantly increased MDA levels as compared with control cells (set 100%). Pretreatment of *T. chebula* extract at 0.1 μg/mL and 1 μg/mL significantly reduced MDA level from 237.0 ± 15.2% in the H_2_O_2_ treated group to 115.8 ± 5.8% (*p* < 0.001) and 93.7 ± 2.2% (*p* < 0.001) respectively (Figure 3B).

### 2.6. Effect of T. chebula Extract in LPS Induced Microglia Cell Activation and NO Inhibition

**Figure 4:**
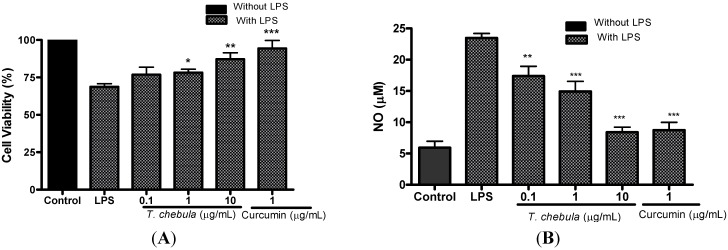
Effect of *T. chebula* extract on LPS-induced cell death and NO inhibition assay. BV2 cells were exposed to LPS (1 μg/mL) for 24 h. Different concentrations of *T. chebula* extract were treated 2 h before and during LPS exposure. (**A**) Cell viability was measured by MTT assay. Control group served as 100%, and data obtained in other groups were calculated as percentage of control accordingly. (**B**) Cell-conditioned supernatants were collected and the production of NO in the supernatants was measured using Griess reagent. NO production was calculated by using standard nitrite. * *p <* 0.05, ** *p <* 0.01, and *** *p <* 0.001.

In order to elucidate the effect of *T. chebula* extract against LPS induced microglia activation, BV2 cells were treated with LPS. Cell viability, after treatment of LPS (1 μg/mL), was found as 68.7 ± 2.0% whereas *T. chebula* extract (at 0.1, 1, and 10 μg/mL) treated cells showed viability of 76.8 ± 4.9%, 78.2 ± 2.2% (*p* < 0.05), and 87.1 ± 4.3% (*p* < 0.01) respectively (Figure 4A). The amount of NO released by BV2 cells was 3.3 ± 0.5 μM in control cells, 19.2 ± 1.1 μM (*p* < 0.001) in LPS treated cells, and 16.6 ± 0.7 μM, 10.0 ± 0.7 μM (*p* < 0.001) and 7.2 ± 0.9 μM (*p* < 0.001) in *T. chebula* extract (0.1, 1, 10 μg/mL) treated cells, respectively (Figure 4B). Curcumin at 1 μg/mL showed 94.3 ± 5.2% (*p* < 0.001) cell viability and 8.7 ± 1.2 μM (*p* < 0.001) NO release in BV2 cells.

## 3. Discussion

In the present study, fruit extract of *T. chebula* significantly reduced PC12 cell death induced by 4 h of OGD followed by 24 h of reoxygenation and 24 h exposure of H_2_O_2_. *T. chebula* extract showed the strong scavenging activity against DPPH free radicals and markedly reduced the H_2_O_2_-induced MDA production. *T. chebula* extract also reduced LPS induced cell death and inhibited the production of NO by BV2 cells in a dose dependent manner.

As OGD-R mimics tissue ischemia, it results in the production of ROS, release from glutamate toxicity, and inflammatory cytokines [25,26]. Generations of the free radicals, such as, superoxide and superoxide-derived oxidants have been invoked as key mediators of tissue injury during ischemia-reperfusion [27]. Cellular injury followed by ischemic reperfusion is a complex pathophysiological event associated with impairment of multiple vascular and cellular responses. Oxidative damage due to the ROS/RNS play the vital role to initiate a wide range of intracellular stress signaling processes that culminate in excessive cytokine and chemokine response, adhesion molecule upregulation, *etc.* [28]. Once the ROS/RNS are produced within the cells, oxidative cascades of events take place simultaneously, including free radical production, sudden start and aggravation of mitochondrial respiratory chains, excitotoxicity, enzymatic changes, and the stimulation of the inflammatory processes [29]. Among such cascades, oxidative stress and inflammation play the key role for the cellular damage. The compounds having antioxidant, anti-inflammatory, anti-glutamate activity show protective activity against hypoxia induced cell damage [26,30,31,32].

*T. chebula* extract significantly protected the PC12 cells against OGD-R. The exposure of PC12 cells to 4 h of OGD followed by 24 h of re-oxygenation resulted decrease in cell viability as compared to control (non-OGD) cells. Incubation of PC12 cells with *T. chebula* extract, 30 min before and during OGD period, led to significant increase in cell viability. It is reported that, OGD mediates the oxidative stress in the cells by activating the different oxidative cascades such as activation of lipoxygenase, IκB kinase, nuclear factor-κB, and OGD-R is the major source of ROS in ischemia [33,34]. In our study, *T. chebula* extract markedly scavenged the nitrogen-centered free radical DPPH (by 96%) in a dose dependent manner, similar to that of ascorbic acid, so the potential nature of antioxidant activity of *T. chebula* extract might be the key factor to sequester the ROS, that are generated in the ischemic cascades, like OGD-R.

The maximum protective effect of *T. chebula* extract (70%) against H_2_O_2_ induced PC12 cell death, was found to be significantly higher than that of the positive control, baicalein (46.3%). H_2_O_2_ is not a free radical and has a limited reactivity; however, it is the major precursor of the highly reactive hydroxyl radical. Previous studies have shown a close association between H_2_O_2_ and neurodegenerative disease, and it has been suggested that H_2_O_2_ levels are increased during pathological conditions, such as ischemia [35,36]. ROS, such as H_2_O_2_, readily damage biological molecules that can eventually lead to apoptotic or necrotic cell death [37]. In our study, interestingly, the extent of cell protection by *T. chebula* extract after H_2_O_2_ induced cell death was higher in less concentration of extract than that in higher concentration (Figure 3A). The mechanism of cell protection by *T. chebula* extract, against H_2_O_2_ induced cell death is largely due to the antioxidant efficacy of polyphenols present in *T. chebula*, such as quercetin, gallic acid and ellagic acids. These polyphenols are reported to show pro-oxidant activity at higher concentrations [38,39]. This might explain the lesser protective effect of *T. chebula* extract at higher concentration (1 μg/mL).

*T. chebula* extract markedly scavenged the nitrogen-centered free radical DPPH in a dose dependent manner. From HPLC analysis, it was observed that main phenolic compounds in *T. chebula* are gallic acid and ellagic acid, which are well-known natural antioxidants [40]. Plant polyphenols are multifunctional in the sense that they can act as reducing agents, hydrogen atom donors, and singlet oxygen scavengers [41]. The DPPH radical scavenging activity of *T. chebula* is mostly related to the hydroxyl group in gallic acid and ellagic acid.

Our results showed that MDA production during H_2_O_2_ treatment was significantly inhibited by *T. chebula*. H_2_O_2_ is believed to be the major precursor for highly reactive free radicals, expressed indirectly by the increase in MDA levels, which is one of the most important organic expressions of oxidative stress in various neuronal diseases including ischemia [42]. Oxidative stress induces cellular damage and lipid peroxidation, which may lead to alterations in membranes and produce significant changes in their biophysical function [43]. Previous studies have shown that the radical scavengers and inhibitors of lipid peroxidation can ameliorate ischemic cell damage [5,44]. Therefore, antioxidant effect through the inhibition of lipid peroxidation and free radical scavenging could be the possible protective mechanisms of *T. chebula* against ischemia and H_2_O_2_ induced cell death.

*T. chebula* extract inhibited the accumulation of NO by 76.2%, at 10 μg/mL. LPS activated BV2 cells release NO in the culture medium which is recognized as a mediator and regulator in pathological reactions, especially in acute inflammatory responses [45]. In addition to ROS, overproduction of NO also plays an important role in various models of inflammation [46,47,48]. The reaction of NO with superoxide anion forms peroxynitrite [49], a potent cytotoxic oxidant eliciting lipid peroxidation and cellular damage [50]. Activated microglia is thought to be involved in neuronal inflammation by overproducing various bioactive molecules such as NO, ROS and pro-inflammatory cytokines [51]. *T. chebula* extract significantly inhibited NO release by LPS activated BV2 cells, elucidating that it can inhibit the activation of microglia. Therefore, anti-inflammatory effect through the inhibition of NO release could be another possible protective mechanism of *T. chebula* against ischemia.

Besides these, *T. chebula* and its active compounds gallic acid and ellagic acids are previously reported to inhibit cytochrome p450 enzyme and protect against mitochondrial dysfunction [52,53]. *T. chebula* treatment significantly increased the level of Cytochrome C oxidase and other mitochondrial enzymes. Gallic acid down-regulates the protein expression and activity of caspase-3, an essential effector molecule in the course of programmed cell death [54]. *T. chebula* is also reported to inhibit inducible nitric oxide synthesis by decreasing iNOS protein and iNOS mRNA levels suggesting the possible intercellular mechanism of *T. chebula* extracts and its active constituents [55]. Taken together, the protective efficacy of the *T. chebula* extract against OGD-R induced PC12 cell death and the inhibition of activated microglia must be due to the antioxidant and anti-inflammatory potential of *T. chebula* extract and its polyphenols.

## 4. Experimental

### 4.1. Plant Material and Chemicals

The fruits of *T. chebula* were collected from Pokhara, Nepal after proper identification by the experts. A voucher specimen (HP203) was deposited in the Herbarium of the Kyung Hee University, College of Oriental Medicine, Seoul, South Korea. Dulbecco’s modified Eagle’s Medium (DMEM), LPS, *Escherichia coli* (0127:138), 3-(4,5-dimethylthiazol-2yl)-2,5-diphenyltetrazolium bromide (MTT), 2,2'-, DPPH, gallic acid, ellagic acid, thiobarbituric acid (TBA), butylated hydroxytoluene (BHT), dimethyl sulfoxide (DMSO) and Griess reagent were all purchased from Sigma Chemical Co. (St. Louis, MO, USA), Fetal Bovine Serum (FBS), penicillin and streptomycin were obtained from Gibco BRL (Grand Island, NY, USA). All reagents and solvents used were of HPLC grade, unless specified.

### 4.2. Extraction of Plant Material

*T. chebula* fruit powder (100 g) was extracted under reflux with 70% methanol (1000 mL). The extract was evaporated with a rotary evaporator under reduced pressure to remove the organic solvent and then lyophilized until dryness. A stock solution of extract was prepared in DMSO and deionized water, respectively. Final DMSO concentration did not exceed 0.1% (v/v) in all treatments.

### 4.3. HPLC Analysis of T. chebula Extract

Methanol extract of *T. chebula* was analyzed by the HPLC with a 600 pump (Waters, Milford, MA, USA) using 250 mm × 4 mm Hypersil^TM^ Gold C18 column (ThermoElectron, Bellefonte, PA, USA), at a flow rate of 1 mL/min under the following conditions: solvent A (0.5% v/v H_3_PO_4_) and solvent B (CH_3_CN); a linear gradient from 5% to 50% v/v of solvent B in A for 60 min. The isolated compounds were monitored with photodiode array detector (926, Waters). Content of major compounds was calculated by comparing with the standard compounds.

### 4.4. Free Radical Scavenging Activity and Total Phenol Content in T. chebula Extract

Free radical scavenging activity was measured spectrophotometrically by DPPH free radical scavenging assay [56] using ascorbic acid as a positive control. Phenolic compounds in the *T. chebula* extracts were determined by the colorimetric assay as described previously [57] with some modifications. Briefly, 1 mL of sample was mixed with an equal volume of Folin and Ciocalteu’s phenol reagent. After 3 min, 1 mL of saturated sodium carbonate solution was added to the mixture. After 90 min of reaction, the optical density (OD) was measured on a plate reader (SPECTRA max Plus^384^, CA, USA) at 725 nm. Gallic acid (10–500 mg/L) was used for calculation, and the results were expressed as mg of gallic acid equivalents (GCE) per gram of extract.

### 4.5. Cell Culture

PC12 cells and BV2 cells were obtained from the Medical Department, Kyung Hee University, Seoul, Korea, and maintained at 37 °C in a humidified atmosphere containing 5% CO_2_. PC12 cells were seeded at a density of 1.5 × 10^3^ cells/well whereas BV2 cells were seeded at a density of 5 × 10^4^ cells/well and cultured at 37 °C in DMEM is supplemented with 10% heat-inactivated FBS, penicillin (1 × 10^5^ U/L) and streptomycin (100 mg/L) in a 5% CO_2_ incubator.

### 4.6. Oxygen-Glucose Deprivation Followed by re-Oxygenation

Oxygen-glucose deprivation followed by re-oxygenation experiments were carried out 24 h after seeding the cells. For the measurement of cytotoxic effects, PC12 cells were treated with *T. chebula* extract (at 0.1, 1, 10 μg/mL) and incubated for 28 h. For OGD injury, PC12 cells were first washed with phosphate buffer saline (PBS, pH 7.2) then with glucose-free DMEM. The cultures were then placed in fresh glucose free DMEM and kept in a hypoxic chamber (Forma Science, UK) containing 95% N_2_ and 5% CO_2_ for 4 h. At the end of the exposure period, glucose solution was added, and the cells were incubated (5% CO_2_ at 37 °C) for an additional 24 h. Samples of different concentrations were treated 30 min before and during OGD exposure. The control group was treated with the same amounts of serum free glucose DMEM and incubated. Baicalein, a potent antioxidative and neuroprotective compound [58] was used as a positive control.

### 4.7. H_2_O_2_ Induced Cell Injury

For H_2_O_2_ treatment, PC12 cells were washed with FBS free DMEM. Dilution of H_2_O_2_ was made from a 30% stock solution into DMEM just prior to each experiment and 200 μM solution added to the cells. The culture plates were incubated for an additional 24 h. Samples of different concentrations were treated 2 h before and during H_2_O_2_ exposure. Control group (without H_2_O_2_) was incubated under the same conditions.

### 4.8. Lipid Peroxidation Assay

Malondialdehyde (MDA), the most abundant lipid peroxidation product from PC12 cells, was measured using the TBA colorimetric assay. Briefly, 24 h after the treatment of the cells with H_2_O_2_ (200 μM) in the presence or absence of *T. chebula* extract (0.1 and 1 μg/mL), cultures were washed with ice-cold PBS, pooled in 0.1 mol/L PBS-5% Triton X-100 buffered solution, and incubated for about 1 h at 37 °C. Trichloroacetic acid (350 μL; 20% w/v) was added to 250 μL of cellular lysate and centrifuged (1,000 × *g* at 4 °C for 10 min). Aliquots (450 μL) of supernatant were mixed with equal volume of 0.5% (w/v) TBA. The mixture was boiled at 100 °C for 30 min. After cooling MDA formation was measured at 520 nm and the results were expressed as a percentage of the control group.

### 4.9. LPS-Induced Microglia Activation and the Measurement of NO

For LPS induced cell death and nitric oxide inhibitory assay of *T. chebula* extract, BV2 cells were incubated with/without 1 μg/mL of LPS, in the absence or presence of different sample solutions for 24 h. For the measurement of NO produced by activated cells, 90 μL of the supernatant from the BV2 cells was mixed with equal volume of the Griess reagent. The OD was measured at 540 nm. Curcumin, a potent anti-inflammatory compound [59] was used as a positive control.

### 4.10. Cell Viability

Cell viability was evaluated by observing the ability of viable cells to reduce yellow colored MTT to purple colored formazan. MTT was dissolved in DMEM and added to the culture at a final concentration of 0.5 mg/mL. After an additional 2 h of incubation, the media was carefully removed and 100 µL DMSO was added to each well. The OD was measured on a plate reader at 570 nm. Results were expressed as a percentage of the control group.

### 4.11. Statistical Analysis

All results are presented as mean ± S.E.M. Significant differences between experimental groups were determined by using one way ANOVA followed by the Tukey’s *post hoc* test using GraphPad Prism 4 (GraphPad Software Inc., La Jolla, CA, USA). *p* < 0.05 is considered as statistically significant for the experimental analysis. Each experiment was performed at triplicate.

## 4. Conclusions

In conclusion, *T. chebula* extract showed a protective effect against OGD-R, H_2_O_2_ and LPS induced cell death. *T. chebula* has an antioxidative and anti-inflammatory activity which could be the possible mechanism for the protection of cells against ischemia. Our results suggest that *T. chebula* fruit could be the useful remedy for ischemia, oxidative stress and activated microglia-induced secondary damage, which occurs in many neurodegenerative diseases. The exact protective mechanism of *T. chebula* extract against OGD-R is unknown, however, it can be estimated that the protective effect of *T. chebula* might be due to its antioxidative and anti-inflammatory effects. Further study is necessary to evaluate the protective effect of *T. chebula* extract on *in vivo* ischemia.
